# ABroAD: A Machine Learning Based Approach to Detect Broadband NIRS Artefacts

**DOI:** 10.1007/978-3-319-91287-5_51

**Published:** 2018-04-16

**Authors:** Joshua Russell-Buckland, Gemma Bale, Isabel de Roever, Ilias Tachtsidis

**Affiliations:** Biomedical Optics Research Laboratory, University College London, London, UK

## Abstract

Artefacts are a common and unwanted aspect of any measurement process, especially in a clinical environment, with multiple causes such as environmental changes or motion. In near-infrared spectroscopy (NIRS), there are several existing methods that can be used to identify and remove artefacts to improve the quality of collected data.

We have developed a novel Automatic Broadband Artefact Detection (ABroAD) process, using machine learning methods alongside broadband NIRS data to detect common measurement artefacts using the broadband intensity spectrum. Data were collected from eight subjects, using a broadband NIRS monitoring over the frontal lobe with two sensors. Six different artificial artefacts – vertical head movement, horizontal head movement, frowning, pressure, ambient light, torch light – were simulated using movement and light changes on eight subjects in a block test design. It was possible to identify both light artefacts to a good degree, as well as pressure artefacts. This is promising and, by expanding this work to larger datasets, it may be possible to create and train a machine learning pipeline to automate the detection of various artefacts, making the analysis of collected data more reliable.

## Introduction

Near infrared spectroscopy (NIRSNear infrared spectroscopy (NIRS) Broadband NIRS)Near infrared spectroscopy (NIRS) instruments use light in the near infrared spectrum (usually only two discrete wavelengths) to measure the changes in haemoglobin concentrations. It is also possible, when using a Broadband NIRS system (more than 100 wavelengths), to observe in addition changes in tissue metabolism via quantification of the oxidative state of cytochrome-c-oxidase [1]. Therefore, with broadband NIRS it is possible to measure changes in Broadband NIRS within the brain, as well as changes in metabolism. This can be important to investigate how the brain responds to stimuli (functional activation) [2] or the impact of injuries such as hypoxic ischaemic encephalopathy [3]. When collecting measurements, external factors (movement, ambient light etc.) can create Artefacts within the data. These can lead to data being less reliable and vary in cause and impact size. We attempt to identify artefacts using Machine learning.

Automatic Broadband Artefact Detection (ABroAD) is the process of identifying patterns within data to try and understand it, preferably in a way that will allow this understanding to be used with new data [4]. Data are normally represented within machine learning as a set of features e.g. number of words in a document or the length of each sentence. Features may already be present in the data or new ones may be engineered from the data available. These data are then used with an algorithm that processes it and produces output such as a Automatic Broadband Artefact Detection (ABroAD) Artefacts or a predicted value. The quality of this prediction is then evaluated using a metric. In the case of a predicted value, that may be its error, or in the case of a classification it may be the classification accuracy or some other suitable metric.

This work aims to develop and use a Automatic Broadband Artefact Detection (ABroAD) platform to identify Broadband NIRS. This is done using the broadband spectra of light rather than the calculated chromophore concentrations. The platform was tested with data generated in a series of experiments wherein subjects simulated artefacts. These data were then used to engineer features that describe each broadband spectrum, before being classified using a random forest classifier.

## Method

Broadband NIRSAutomatic Broadband Artefact Detection (ABroAD) data were collected from eight different subjects in a block test design using a Custom-built broadband NIRS system Broadband NIRS, based on a system previously described by Bale et al. [3], at a sample rate of 5 Hz. Two sensors were used: a short separation: sensor 13 at 10 mm, and a long-separation: sensor 7 at 30 mm. Six different artefacts were simulated – horizontal motion (shaking head), vertical motion (nodding head), frowning, pressure on sensor, ambient room light and directed torch light – in 10 s blocks repeated twice. There was 10 s of rest between each artefact, leading to roughly 50 data points for each block.

The start and end of each artefact, as well as the start and end of the experiment, was marked in the output data as an event using the LabVIEW software which collects data from the NIRS system. All artefacts were simulated in the order listed above for all subjects.

**Fig. 1:**
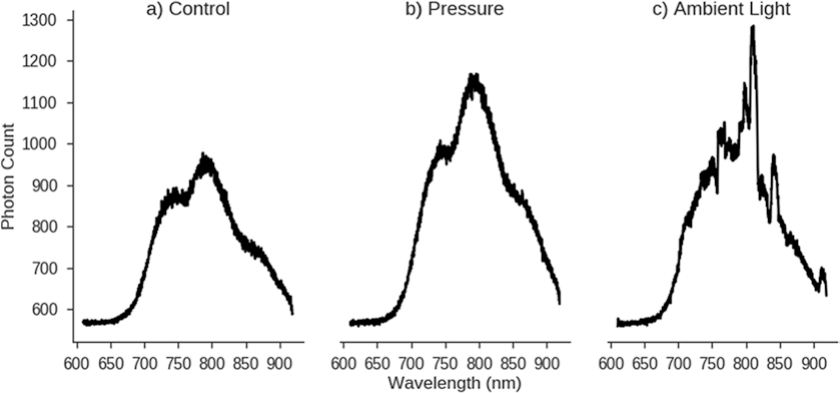
Example spectra (integration time 0.2 s) from subject 5 during control (**a**) and two Artefacts (**b**, **c**)

At each time point a spectrum of light was collected for 1340 wavelengths between 610 and 920 nm, as seen in Fig. 1. Thus, these data have an extremely high dimensionality and can also be deemed to be functional, i.e. each wavelength is functionally related to its neighbouring wavelengths. Many machine learning approaches assume data points to be independent so, to both reduce the dimensionality and generate features that are not functionally related, feature engineering was undertaken.

Feature engineering describes the process by which new featuresNear infrared spectroscopy (NIRS) Artefacts are generated from existing data. The features were chosen by looking at different spectra for each artefact and attempting to identify differences that could then be summarised in a single number or measureAutomatic Broadband Artefact Detection (ABroAD). These were: **power density fraction**, **sample entropy**, **autocorrelation** and **area under the curve.**

**Fig. 2:**
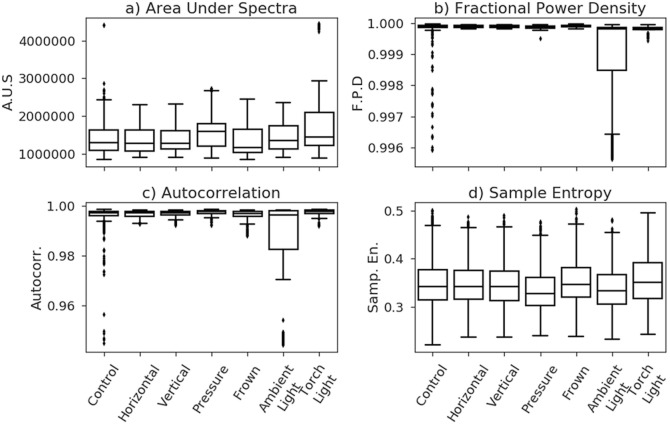
Distribution of feature values for each artefact

The ambient light artefact is one of the most noticeable, as the fluorescent lights used in the room led to spikes in intensity at specific wavelengths, as seen in Fig. 1c. It was found that the fraction of the integrated power density spectrum occupied by the top 99% of frequencies (referred to as the Automatic Broadband Artefact Detection (ABroAD) Fractional power density) was generally lower in the spectra containing ambient artefacts as compared to a control. Figure 2a shows there is a clear difference between distributions when considering the ambient light artefact compared to all others. The torch light artefact also shows a distribution of values that are clearly separate compared to all non-light artefacts, though to a much lesser degree.

Automatic Broadband Artefact Detection (ABroAD) Sample entropy is a modification of approximate entropy, chosen due to it being more computationally efficient, and is a measure of the complexity level within a signal [5]. Figure 2b shows the distribution of sample entropy values. The control, horizontal and vertical artefacts show little difference, but both pressure and ambient light have distributions that are generally lower than the other artefacts, whilst torch light appears to have a distribution that is generally higher.

Automatic Broadband Artefact Detection (ABroAD) Autocorrelation is the correlation of a signal with a time delayed copy of itself as a function of delay. Figure 2c shows the distribution of autocorrelation values for each artefact, where the ambient light artefact shows a markedly different distribution to the other artefacts.

Many artefacts, particularly those due to changes in light, showed an increase in intensity for many wavelengths, increasing the area under the curve. This could be calculated by Automatic Broadband Artefact Detection (ABroAD) Integrating under the light spectrum using the trapezoidal rule. Good separation can be seen in Fig. 2d, particularly in the pressure artefact and both light artefacts.

**Fig. 3:**
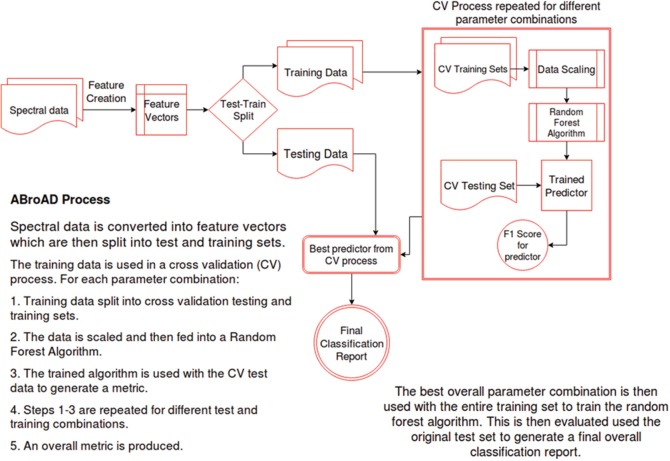
Outline of the Machine learning process

Figure 3 outlines the Automatic Broadband Artefact Detection (ABroAD). For each subject, *i*, the spectrum at each time point, t_j_ is converted into a four-dimensional feature vector, **x**_i,j_ of the form {*x*_*i*, *j*, 1_, *x*_*i*, *j*, 2_, *x*_*i*, *j*, 3_, *x*_*i*, *j*, 4_} and assigned a true classification y_i,j_ according to the artefact simulated at that time point. This dataset is split into test and training sets, based on the subject number, *i*. This ensures that the algorithm is tested on data from an unseen subject.

The training data are then fed into a machine learning pipeline consisting of two main steps: Automatic Broadband Artefact Detection (ABroAD). Scaling is done using the ‘RobustScaler’ from the Scikit-learn library [6] and is done to ensure all features are roughly equatable in terms of magnitude. Without this, features that are of significantly different magnitudes to others, e.g. AUC, may receive undue weighting in the estimation process. The scaled data are then passed into a random forest classifier [7].

This is fitted using a Automatic Broadband Artefact Detection (ABroAD). The training data are split by subject into training and test sets “M times”, with M = 10, allowing retesting of the Automatic Broadband Artefact Detection (ABroAD) of the overall training set. The classifier is run for different parameter combinations, and the set that provides the best final score is chosen as the best estimator. This is then trained on the total training set and tested on the initial test setNear infrared spectroscopy (NIRS) Artefacts. This final score allows the effectiveness of the chosen method to be evaluated.

The Automatic Broadband Artefact Detection (ABroAD) chosen here is the `weighted F1-score’ – where a perfect classification has a score of 1 and no correct classifications would have a score of 0 – which accounts for both precision (p) and recall (r) and is able to deal with the class imbalance inherent in the data. It is defined as F1 = 2*p*r/(p+r). Precision is the fraction of correct classifications for a class *j* out of the total number of predictions of that class, whilst recall is the fraction of correct classifications for a class *j* out of the total number of actual occurrences of that class.

Initially, all Automatic Broadband Artefact Detection (ABroAD) were considered together. A distinct difference can be seen between the F1-scores for light artefacts and for motion artefacts. Therefore, it was decided to additionally consider datasets that contained just motion artefacts and just light artefacts to determine if classification could be improved by doing so.

## Results

**Fig. 4:**
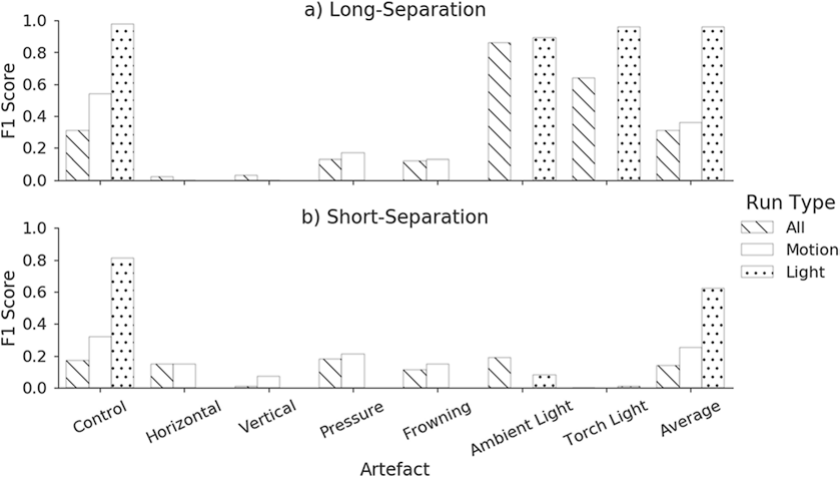
F1-scores for each artefactArtefacts and run type

Automatic Broadband Artefact Detection (ABroAD) were selected randomly, splitting by subject. The same test and training splits were used for all results. The algorithm was trained using data from subjects 1, 2, 3, 4, 6 and 8 and tested against data from subjects 5 and 7. Figure 4a, b show the F1-scores for each artefact, type of model run and for each sensor. The Automatic Broadband Artefact Detection (ABroAD) shows a much better set of light only scores than the short-separation (**ambient**: 0.89 vs 0.08, **torch**: 0.96 vs 0.01). Across both sensors there is a clear inability to detect motion artefacts, but this is improved by omitting light artefacts when classifying. Additionally, for both sensors the algorithm is able to detect the non-presence of an artefact when only considering light artefacts.

## Discussion

We have developed a Broadband NIRS. We have shown that it can detect the non-presence of light artefacts across both long and short sensors, as well as the ability to determine the presence of specific light artefact types in the long-distance sensor.

The algorithm shows difficulty Detecting motion artefacts, particularly those due to horizontal and vertical movement. This may be because the choice of engineered features does not adequately capture information that can distinguish these artefacts, or it may be that these artefacts were not adequately simulated during data collection. Double cross-validationNear infrared spectroscopy (NIRS) Artefacts [8] will be used to further validate this process, ensuring that test scores are not test set dependent.

New features can be easily added into the process and, with further data collection, the platform can be improved to detect these artefacts. Additionally, whilst the data used here are from a broadband NIRS system, there is the potential for data from accelerometers and external light sensors to be used with the platform where broadband data are not available.
